# At slaughtering and post mortem characteristics on Traditional market ewes and Halal market ewes in Tuscany

**DOI:** 10.1186/s40781-016-0116-2

**Published:** 2016-09-07

**Authors:** Clara Sargentini, Roberto Tocci, Matteo Campostrini, Eleonora Pippi, Valeria Iaconisi

**Affiliations:** Dipartimento di Scienze delle Produzioni Agroalimentari e dell’Ambiente (DISPAA) – sez. Scienze Animali, Università degli Studi di Firenze, Via delle Cascine, 5, 50144 Firenze, Italy

**Keywords:** Ewe, Traditional market, Halal market, Carcass characteristics, Meat quality, PCA analysis, Consumer test

## Abstract

**Background:**

The aim of this work was the comparison between the carcass and the meat ewes of the regional Traditional market and the Islamic religious (Halal) market.

**Methods:**

Thirty and 20 at the end of career traditional market and Halal market ewes were slaughtered following the EC (European Council, 2009) animal welfare guidelines. Live weight of ewes was taken and dressing percentage of carcasses was calculated. On every carcass zoometric measurement and the evaluation trough the EU grid rules were performed. On the *Musculus longissimus thoracis* of 12 Traditional market carcasses and 11 Halal market carcasses the physical-chemical and nutritional analysis were performed. Consumer tests for liking meat ewe were performed in order to find consumer’s preference level for Traditional and Halal markets ewe meat. Considering as fixed factor the ewe meat market (Traditional and Halal), results were submitted to oneway Analysis of Variance (ANOVA) and to Principal Component Analysis (PCA).

**Results:**

The Halal market ewes have shown lower dressing percentages (42.91 ± 0.82 vs 46.42 ± 0.69) and lower conformation score (4.5 ± 0.5 vs 7.8 ± 0.4). The Halal market meat showed higher cooking loss in oven (37.83 ± 1.20 vs 32.03 ± 1.15 %), lesser Chroma value (18.63 ± 0.70 vs 21.84 ± 0.67), and lesser Hue angle value (0.26 ± 0.02 vs 0.34 ± 0.02). This product had also lower fat percentage (4.2 ± 0.4 vs 7.09 ± 0.4). The traditional market meat had higher percentage in monounsatured fatty acids (MUFA) (43.84 ± 1.05 vs 38.22 ± 1.10), while the Halal market meat had higher percentage in ω3 poliunsatured fatty acids (PUFA) (5.04 ± 0.42 vs 3.60 ± 0.40). The consumer test showed as the ewe meat was appreciate by the consumers.

**Conclusions:**

Both meat typologies have shown good nutritional characteristics. The traditional market meat had higher MUFA composition, and a better MUFA/satured fatty acids (SFA) ratio, while the Halal market meat had higher PUFA composition. These results were also supported by the PCA. The consumers preferred the traditional market meat.

## Background

The more consumed ovine meat in Tuscany is the lamb meat, in particular during the Christmas and the Easter time. In this region the 78 % of raised sheep derives from dairy breeds, mainly from Sarda sheep; in the 2010, 343.375 sheep were slaughtered, and of these, 87 % were 8–12 kg live weight suckling lambs, 11.9 % ewes and muttons and only 0.8 % heavy lambs, and castrated [1]. The ewe meat is ever available on the market, because it derives from at the end of career sheep or discarded reproducers; this meat is not appreciated in the great part of Tuscany, and only in some areas of Firenze, Prato, and Pistoia provinces the ewe meat is eaten. Among the immigrating populations, Islamic peoples and Eastern-European peoples require this product [2]. For the northern-African populations the highest request is at the end of the religious Holidays (Ramadan). During the year, the Halal carcasses quality is generally not excellent, and the carcasses derive from emaciated and not conformed animals. The meat of these animals is cheap, and it’s required for tajines and typical dishes preparation. In the Tuscan butcher’s shops an average of 203 lambs, and a very low number of sheep, are sold every year. In the Halal butcher’s shop an average of 153 ewes are sold in a year [2]. The Halal meat in Tuscany derives from sheep slaughtered following the Animal Welfare European Union (EU) Regulation 1099/2009 guidelines [3], which provide the stunning before the slaughtering. The traditional and the Halal slaughtering are not different, except for small differences: the ritual method must to follow some laws; the name of Allah must be invoked by saying: Bismillah Allahu Akbar, the head of the animal must not be cut off during slaughtering but later after the animal is completely dead, any instrument used for slaughtering pigs should not be used in the Halal slaughtering, etc. [4]. The aim of this work is to compare the ewe meat deriving from the Traditional and the Halal butcher ‘shops.

## Methods

### Animal welfare

The ewes of this trial were slaughtered following the Council Regulation of 24 September 2009 on the protection of animals at the time of killing - animal welfare guidelines, which provide the head-only electrical as stunning method (European Council 2009. Council regulation (EC) No 1099/2009 of 24 September 2009 on the protection of animals at the time of killing (Text with EEA relevance) [3].

### Animals and carcass traits

In this work the post mortem preliminary results on 30 ewes deriving from the Tuscan traditional market and 20 ewes deriving from the Tuscan Halal ovine market were reported; all animals were reared in semi-extensive conditions and were at the end of career. The ewes of this trial, were slaughtered at the Lanini slaughterhouse, in Arezzo province (Castel San Niccolò), following the Council Regulation of 24 September 2009 on the protection of animals at the time of killing - animal welfare guidelines, which provide the head-only electrical as stunning method [3]. The recommended parameter for sheep and goats are: minimum level of 1 A of power [5]. The head-only electrical stunning duration was 5 s, ensuring the animal pain sensation lose before the slaughtering [5]. The slaughtered animals were chosen considering the features of the ewes usually intended for the traditional and the Halal markets. The owner of the slaughterhouse chosen the ewes intended for the traditional market, while the Islamic slaughterer chosen the ewes intended for the Halal market. The chosen ewes for the traditional market ranged in age from 3 to 7 years, and belonged to the following local sheep breeds and crosses: Appenninica x Sarda (9), Sarda (14), Comisana (7). The chosen ewes for the Halal market were over 7 years old, and belonged to the following local sheep breeds and crosses: Sarda (12), Appenninica x Sarda (8). The Appenninica sheep is a medium-large meat purpose breed, Sarda sheep is a small dairy breed, and Comisana sheep is a medium-large dairy breed. The 53 % of the total slaughtered ewes for the traditional market were medium-heavy, while only 35 % of the total slaughtered ewes for the Halal market ewes had this size.

In all ewes, the live weight was taken. After the slaughtering, the internal organs (heart, trachea, lungs, spleen, liver, rumen, reticulum, omasum, abomasum, large and small intestines) weight and the slaughtering discarded (head, skin, legs) weight were taken. On the carcasses the weight, and the zoometric measurements [6] were taken. The dressing percentage was calculated. The carcasses were evaluated for conformation and fat score following EU grid rules (15 points scale) [6].

### Laboratory analysis

After a 4 days at 4 °C refrigeration, from 12 traditional market carcasses and 11 Halal market carcasses, the right *Musculus longisssimus thoracis* was taken, in order to determine the physical-chemical and nutritional characteristics. The physical parameters were the following: water holding capacity, determined either as drip loss or as cooking loss in water bath [7] and in oven [8], the free water, determined through the Grau and Hamm method [9]; meat colour was determined with a Minolta Chromameter CR 200 (CIE L, a*, b*). Chroma (colour saturation – (a2+ b2)1/2) and Hue angle (arctan b/a) were also calculated [7]. Texture analyses [10] in raw and in water bath meat were carried out using a Zwick Roell® 109 texturometer (Ulm, Germany) with Text Expert II software, equipped with a 1 kN load cell. The Warner-Bratzler shear test (WB-shear force) was performed using a straight blade (width of 7 cm), perpendicular to the muscle fibre direction, at a crosshead speed of 30 mm/min to 50 % of total deformation. Maximum shear force, defined as maximum resistance of the sample to shearing [11] was determined. Chemical analyses were carried out on each sample of muscle determining dry matter, fat (ether extract), crude protein and ash [12]. The samples were also analysed for total lipid concentration by gravimetric determination of total lipid extract according to Folch et al. [13] and for quantitative fatty acid composition of total lipids by gas chromatographic separation of methyl esters, comprising C19:0 as internal standard, on capillary column oven temperature ranging from 164 to 200 °C with 3 °C/min heat increment. Atherogenic (AI) and Thrombogenicity (TI) Indexes were calculated according to Ulbricht and Southgate [14].

### Consumer test

On the traditional and Halal markets meat a consumer test was performed in order to evaluate the ewe meat liking level. One-hundred-seventy-eight Christian usual meat consumers, but not usual ewe consumers, participated to the consumer test, previously responding about the ewe meat expectations, and at the end of the test responding about the ewe meat overall liking level. The answers had a grading scale from 1 to 9 (1 = extremely negative.... 9 = extremely positive). Two identical salt and pepper steaks, one from the traditional market ewe loin and one from the Halal market ewe loin, slaughtered for the trial in the Lanini slaughterhouse, were brought together to the consumers and identified with the letters A and B respectively. For every steak the consumers have given a vote (from 1 to 9) for the taste, odour, tenderness, and the overall liking.

### Statistical analysis

Data were submitted to GLM oneway ANOVA with JMP 10 [15] considering as variability factor the product market destination (traditional and Halal). For the fatty acids composition, PCA was applied. PCA belongs to the group of multivariate analysis methods. Its basic concept is to describe a given phenomenon using a small number of so-called hidden factors (i.e., component) in relation to an extensive set of primary variables. This specific method was selected to determine the degree of similarity between the fatty acids composition in the traditional and Halal markets meat ewe. The Eigenvalue, the cumulative percentage of variance and the Bartlett test was applied, and to maximise the variance of the loadings on the factors, Varimax rotation was applied [16]. A Biplot graphics was also performed in order to visualise the fatty acids distribution in the traditional market meat and in the Halal market meat.

## Results and discussion

### Live animals and carcass traits

The Halal market ewes live weight was lower than that of the traditional market sheep (Table 1). This result was due to the Islamic slaughterers and consumers choices, which prefer small ewes as highlighted in a previous territorial survey. For the Traditional market, mainly large breed sheep (e.g. Appenninica sheep and crosses) were chosen, while for the Halal market mainly small dairy breed sheep (e.g. Sarda sheep and crosses) were chosen. The traditional market ewes were also more conformed, and healthier than those of the Halal market. The carcass weight and the dressing percentage were higher in the traditional market ewes.

**Table 1:** Traditional and Halal markets ewes dressing percentage (mean ± SEM)

		Traditional market	Halal market
Live weight	Kg	49.016 ± 1.424 A	38.238 ± 1.702 B
Carcass weight	Kg	22.727 ± 0.743 A	16.486 ± 0.888 B
Dressing percentage	%	46.42 ± 0.69 A	42.91 ± 0.82 B
Gastrointestinal content weight	Kg	6.551 ± 0.250	6.290 ± 0.422
Net live weight	Kg	42.465 ± 1.311 A	32.518 ± 2.568 B
Net Dressing percentage	%	53.75 ± 0.86 A	50.52 ± 1.02 B

A, B: *P* < 0.05

No significant differences were shown in the internal organs and in the discarded percentage between ewe markets (Table 2), to the exclusion of the offal and the lungs + trachea percentages, higher in the Halal market ewes. In these ewes the offal percentage was enough 8 % of the live weight, and this result was due to the high lung and trachea percentage, that was enough double respect the traditional market ewes. Important loses were represented by the skin, and the head. The carcass measurements (Table 3) have shown similar characteristics of Mediterranean area mesomorphic ewes [17].

**Table 2:** Percentage on net live weight of internal organs and discarded of Traditional and Halal markets ewes (mean ± SEM)

		Traditional market	Halal market
Net live weight	Kg	42.465 ± 1.311 A	32.518 ± 1.567 B
Right front shank	%	0.59 ± 0.08	0.65 ± 0.02
Right hind shank	%	0.60 ± 0.023 B	0.73 ± 0.03 A
Skin	%	9.15 ± 0.30	9.73 ± 0.36
Head	%	6.89 ± 0.15	7.08 ± 0.18
Offal	%	6.18 ± 0.27 B	8.24 ± 0.31 A
Lungs + trachea	%	2.38 ± 0.21 B	4.43 ± 0.24 A
Heart	%	0.87 ± 0.02	0.81 ± 0.03
Spleen	%	0.43 ± 0.02	0.46 ± 0.03
Liver	%	2.59 ± 0.09	2.53 ± 0.10
Stomachs	%	6.53 ± 0.17	6.15 ± 0.21
Intestines	%	7.27 ± 0.25	6.51 ± 0.30

A, B: *P* < 0.05

**Table 3:** Traditional and Halal markets ewe carcass measurement (mean ± SEM)

		Traditional market	Halal market
Carcass length	cm	73.2 ± 1.2 A	69.2 ± 1.4 B
Trunk length	cm	42.3 ± 1.0	39.5 ± 2.7
Thigh length	cm	39.0 ± 0.8	40.8 ± 1.0
Ramp width	cm	28.4 ± 0.4 A	26.6 ± 0.5 B
Thorax width	cm	25.4 ± 0.5	24.2 ± 0.6
Thorax depth	cm	27.2 ± 0.3 B	29.2 ± 0.5 A

A, B: *P* < 0.05

The Traditional market carcasses, with higher carcass length and ramp width, were larger and more conformed than the Halal carcasses. The higher thorax depth of the market Halal carcasses confirmed the higher lungs and trachea percentage previously observed in these ewes.

The traditional market carcasses were more conformed (Table 4). Similar score (R) for the carcass conformation was shown in Hungarian merino x Ile de France ovine crosses [18]. The Halal market carcasses, mainly deriving from the Sarda sheep, showed for the conformation, lower values than the Sarda sheep carcasses studied by Mazzette et al. [19], while the fat score was similar.

**Table 4:** Traditional and Halal markets ewe carcass - EU grid rules carcass evaluation (mean ± SEM)

	Traditional market	Halal market
Conformation score		
(S)EUROP - UE	R	O-
15 points scale, ASPA, 1991	7.8 ± 0.4 A	4.5 ± 0.5 B
Fat score		
1–5 - UE	2+	2+
15 points scale, ASPA, 1991	6.1 ± 0.4	6.1 ± 0.5

A, B: *P* < 0.05

### Physical characteristics of the meat

In comparison with the traditional market meat, the Halal market meat have shown higher water content, with lower red index (a*) and yellow index (b*), and lower Choma (C*) and Hue angle (h*) values (Table 5). These results confirmed the Halal traders and consumer choices, which prefer lesser coloured meat [2]. The colour parameters, in particular redness, chroma and hue angle, were higher in the traditional market ewe meat, probably because these animals were younger; myoglobin loses its affinity for oxygen as age increases [20]. The Halal market meat L* and a* values were higher than those of the Sarda sheep meat [19], while b*, C*, and h* values were similar. No differences were found for the shear force in both meat typologies, and this parameter was higher than that of Merino ewes X Domer and Suffolk rams meat [21].

**Table 5:** Traditional and Halal markets ewe meat physical analysis (mean ± SEM)

		Traditional market	Halal market
Water holding capacity			
Cooking loss in oven	%	32.03 ± 0.15 B	37.83 ± 1.20 A
Cooking loss in water bath	%	42.74 ± 3.24	39.73 ± 3.39
Drip loss	%	4.67 ± 0.59	3.34 ± 0.62
Free water	cm^2^	11.3 ± 0.5	12.7 ± 0.5
Tenderness			
Shear force in cooked water bath meat	N	87.29 ± 12.45	115.62 ± 13.64
Shear force in cooked water bath meat	Kg	8.90 ± 1.27	11.79 ± 1.39
Colour			
L*		38.05 ± 1.11	39.26 ± 1.16
a*		20.57 ± 0.70 A	17.98 ± 0.73 B
b*		7.26 ± 0.49 A	5.05 ± 0.51 B
C*		21.84 ± 0.67 A	18.63 ± 0.70 B
H*	rad	0.34 ± 0.02 A	0.26 ± 0.02 B
H*	°	19.40 ± 1.35 A	15.00 ± 1.41 B

A, B: *P* < 0.05

### Chemical characteristics of the meat

In Table 6 the lean meat chemical composition was shown. A higher dry matter and fat percentage in the traditional market meat was shown; this result could be due to the Halal market ewes characteristics, which were older and emaciated.

**Table 6:** Traditional and Halal markets ewe lean meat chemical analysis (mean ± SEM)

		Traditional market	Halal market
Dry matter (d.m.)	%	27.30 ± 0.54 A	23.55 ± 0.56 B
Moisture	%	72.70 ± 0.54 B	76.45 ± 0.56 A
Ashes	%	1.26 ± 0.08	1.15 ± 0.09
Crude protein	%	18.81 ± 0.37	19.30 ± 0.39
Fat	%	7.09 ± 0.4 A	4.2 ± 0.4 B

A, B: *P* < 0.05

### Fatty acids composition and healthy indices of the meat

In Table 7 the fatty acids composition in the Traditional and Halal markets meat was shown. Both meat typologies were healthy: the traditional market meat had higher MUFA percentage and a better MUFA/SFA ratio, while the Halal market meat had higher ω3 and ω 6 PUFA percentage. The Halal meat mainly derived from dairy sheep that need high grass percentage in the diet [22]: this allows to a higher PUFA percentage in the meat [23].

**Table 7:** Fatty acids on total lipids in Traditional and Halal markets ewe meat (mean ± SEM)

		Traditional market	Halal market
Satured Fatty Acids SFA	%	44.59 ± 0.65	46.53 ± 1.10
Monounsatured Fatty Acids MUFA	%	43.84 ± 1.05 A	38.22 ± 1.10 B
Polyunsatured Fatty Acids PUFA	%	11.46 ± 0.98 B	15.24 ± 1.22 A
PUFA ω3	%	3.60 ± 0.40 B	5.04 ± 0.42 A
PUFA ω6	%	7.86 ± 0.67 B	10.24 ± 0.7 A
MUFA/SFA		0.98 ± 0.03 A	0.82 ± 0.03 B
PUFA/SFA		0.25 ± 0.02	0.33 ± 0.02
ω6/ω3		2.37 ± 0.18	2.08 ± 0.19
ω3/ω6		0.46 ± 0.04	0.50 ± 0.04
Atherogenic Index AI		0.51 ± 0.03	0.59 ± 0.03
Thrombogenic Index TI		0.50 ± 0.02	0.46 ± 0.02

A, B: *P* < 0.05

The health lipids indices, as the ω3/ω6 ratio, the TI and the AI indices didn’t show differences between products. The health lipids indices of the Halal market meat had similar values of those of the adult (between 2 and 7 y old) Sarda ewe meat [24].

The Traditional market meat had a higher anteiso-heptadecanoic acid (Margaric acid) composition (Table 8), an anti-cancerogenous ramificated fatty acid [25]. This meat had also a higher Oleic acid (C18:1 cis9) composition, a healthy fatty acid having cholesterol-decreasing effect [26]: this fatty acid increases the blood cholesterol HDL (High-density lipoprotein), that increases the cholesterol solubility and decreases the atheromatous plaques formation [27]. The Halal market meat had higher myristic acid percentage (C14:0), having unhealthy effect on the cardio-circulatory system [28], but this meat had also higher ω6 and in particular ω3 fatty acids composition, having healthy effect on the cardio-circulatory system [29, 30].

**Table 8:** Fatty acid percentage on total lipids in Traditional and Halal markets ewe meat (mean ± SEM)

	Traditional market	Halal market
C12:0	0.07 ± 0.003	0.17 ± 0.003
C13:0	0.011 ± 0.01	0.01 ± 0.001
C14:0	1.79 ± 0.30 B	2.77 ± 0.31 A
C14:0 iso	0.06 ± 0.006	0.06 ± 0.066
C14:1 n5	0.05 ± 0.01	0.07 ± 0.01
C15:0	0.48 ± 0.03	0.54 ± 0.03
C15:0 iso	0.15 ± 0.01	0.17 ± 0.01
C15:0 ai	0.18 ± 0.02	0.23 ± 0.02
C16:0	22.7 ± 0.62	22.4 ± 0.65
C16:0 iso	0.20 ± 0.01	0.19 ± 0.01
C16:1 n9	0.37 ± 0.01	0.34 ± 0.01
C16:1 n7 cis	1.37 ± 0.08	1.45 ± 0.08
C17:0	1.21 ± 0.04	1.08 ± 0.04
C17:0 anteiso	0.66 ± 0.02 A	0.55 ± 0.02 B
C18:0	16.96 ± 0.84	18.24 ± 0.88
C18:1 n9	37.04 ± 1.26 A	29.88 ± 1.31 B
C18:1 n7	2.74 ± 0.19	2.76 ± 0.20
C18:2 n6 cis	4.49 ± 0.48	6.00 ± 0.50
C18:3 n3	1.49 ± 0.17	1.68 ± 0.18
C20:0	0.10 ± 0.009	0.12 ± 0.009
C20:1 n9	0.20 ± 0.01	0.21 ± 0.01
C20:2 n6	0.08 ± 0.01 B	0.19 ± 0.01 A
C20:3 n6	0.05 ± 0.01 B	0.15 ± 0.01 A
C20:3 n3	0.02 ± 0.0002	0.02 ± 0.002
C20:4 n6	0.67 ± 0.17 B	1.62 ± 0.18 A
(ETA) C20:4 n3	0.01 ± 0.002 B	0.03 ± 0.003 A
(EPA) C20:5 n3	0.19 ± 0.06 B	0.60 ± 0.006 A
C22:0	0.001 ± 0.003	0.02 ± 0.003
C22:1 n9	0.05 ± 0.002 A	0.01 ± 0.002 B
C22:4 n6	0.03 ± 0.005 B	0.05 ± 0.005 A
C22:5 n 3	0.26 ± 0.06 B	0.59 ± 0.06 A
C22:6 n3	0.08 ± 0.02 B	0.16 ± 0.01 A

A, B: *P* < 0.05

### PCA analysis on fatty acids composition of the meat

The PCA identified 6 significant components at Bartlett test (Table 9): these first six components covered enough 87 % of the total variability, constituted by 28 parameters concerning the fatty acids composition in the Traditional and Halal markets meat. Over the 6, the components eigenvalues were lower than 1, and were not significant for the interpretation of PCA results [31]. The PC1 covered almost 40.0 % of the variability, while the PC2 covered 18 %.

**Table 9:** Eigenvalue, cumulative percentage of variance and Bartlett test

	Eigenvalue	Percent	Percentage	Cumulative %	Chi-quare	DF	Prob > ChiSq
1	11.16	39.88		39.88	534.12	374.45	<0.0001*
2	5.09	18.18		58.05	380.98	367.88	0.31
3	3.14	11.22		69.27	288.35	349.55	0.99
4	2.60	9.31		78.58	219.41	328.30	1.00
5	1.21	4.32		82.91	149.01	306.63	1.00
6	1.14	4.09		87.001	114.67	283.64	1.00
7	0.95	3.40		90.40	77.83	261.51	1.00
8	0.72	2.58		92.98	42.99	239.85	1.00
9	0.48	1.72		94.71	13.84	218.95	1.00
10	0.42	1.49		96.20	.	198.64	.
11	0.31	1.12		97.33	.	179.46	.
12	0.26	0.93		98.26	.	161.02	.
13	0.18	0.67		98.94	.	143.46	.
14	0.12	0.44		99.38	.	127.08	.
15	0.09	0.33		99.71	.	111.39	.
16	0.05	0.19		99.91	.	96.66	.
17	0.02	0.10		100.01	.	83.06	.
18	0.02	0.07		100.08	.	70.35	.
19	0.01	0.05		100.13	.	58.41	.
20	0.01	0.04		100.18	.	47.73	.
21	0.008	0.03		100.20	17.28	38.13	0.99
22	0.001	0.005		100.21	49.84	29.82	0.012*
23	0.0008	0.003		100.21	84.69	22.16	<0.0001*

*P < 0.05

In Table 10 the component loading matrix after VARIMAX rotation was shown. Factor 1 identified 17 fatty acids: three SFA, four MUFA and ten PUFA of which five ω3 PUFA. Factor 2 identified three SFA and three MUFA.

**Table 10:** Component loading matrix after Varimax rotation determined for intramuscular lipids fatty acids composition in traditional and halal ewe meat

	Factor 1	Factor 2	Factor 3	Factor 4	Factor 5	Factor 6
C14:0		0.90				
C14:1		0.95				
C15:0	0.43		0.66			−0.003
C16:0	−0.53	0.52	−0.57			
C16:1				−0.83		
C16:1-n7		0.91				
ai-C17:0	−0.58		0.75			
C17:0			0.89			
C17:1	0.67		0.43			0.36
C18:0		−0.68		0.43	0.45	
C18:1-n9	−0.70			−0.55		0.09
C18:1-n7			0.49	0.56	0.34	
C18:2-n6	0.91					
C18:3-n4			0.65	0.49		
C18:3-n3	0.45			0.74		
C20:0				0.33	0.57	0.46
C20:1-n9		−0.42		0.56		
C20:1-n7	0.52				0.38	
C20:2-n6	0.71				0.56	
C20:3-n6	0.94					
C20:4-n6	0.97					
C20:4-n3	0.76			0.52		
C20:5-n3	0.81			0.49		
C22:1-n11						0.94
C22:1-n9	0.54				0.57	
C22:4-n6	0.85					
C22:5-n3	0.92					
C22:6-n3	0.85					

**Fig. 1:**
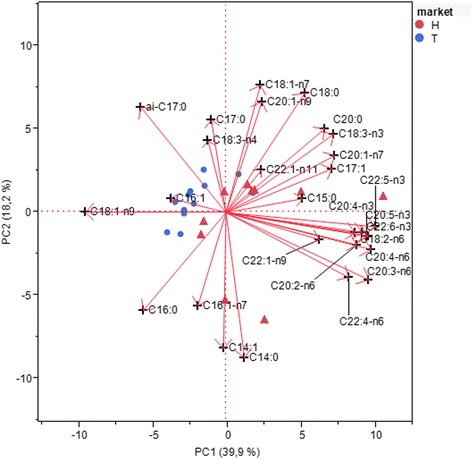
Rohlf Biplot for standardized PCA of fatty acids composition in traditional and Halal adult ovine meat

The Rohlf Biplot graphic (Fig. 1) has shown that the traditional market meat was identified by the Oleic acid and by MUFA, which in ANOVA were in higher percentage. PUFA identified the Halal market meat.

### Consumer test

**Fig. 2:**
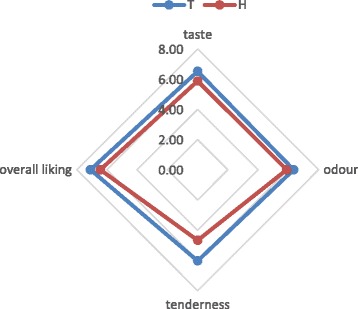
Traditional and Halal markets ewe steak evaluation (final vote)

Concerning the ewe meat liking, the consumers positive expectations (score 7.1 ± 1.53) were confirmed by the high final vote (7.5 ± 1.06). In Table 11 and in Fig. 2 the ewe steak liking evaluation was shown: the traditional market steak was more appreciate in all considered parameters than the Halal market steak.

**Table 11:** Traditional and Halal markets ewe steak evaluation (final vote)

	Traditional market	Halal market
Taste	6.51 ± 0.14 A	5.87 ± 0.14 B
Odour	6.35 ± 0.15 A	5.88 ± 0.15 B
Tenderness	6.03 ± 0.16 A	4.67 ± 0.16 B
Overall liking	6.95 ± 0.13 A	6.37 ± 0.07 B

A, B: *P* < 0.05

## Conclusions

Experimentation results have shown that the traditional market ewes had a better conformation than the Halal market ewes. The Halal market ewes, small and with poor conformation, have shown developed transversal diameters (ramp width) and lower dressing percentage than the Traditional market ewes. The Halal market meat had a higher cooking loss in oven. The Halal market meat, rose-coloured, meted the Islamic consumers preferences.

The lean meat had higher water content, and lesser fat content in the Halal market meat.

Good nutritional characteristics in both meat typologies were shown: the traditional market meat had higher MUFA percentage, and a better MUFA/SFA ratio, while the Halal market meat had higher PUFA percentage, especially ω3 PUFA. These results were also supported by the PCA. The ewe meat (from Traditional and from Halal market) physical-chemical and organoleptic parameters have shown good characteristics. These results can promote the ewe meat consumption in Tuscany, where the sheep main product is the suckling lamb.

## Abbreviations

a*: Redness
AI: Atherogenic index
ANOVA: Analysis of variance
b*: Yellowness
C*: Chroma
EU: European Union
H*: Hue angle
L*: Lightness
MUFA: Monounsatured fatty acids
PCA: Principal component analysis
PUFA: Poliunsatured fatty acids
SFA: Satured fatty acids
TI: Thrombogenicity index
